# 4-Ethoxy­pyridin-2-amine

**DOI:** 10.1107/S1600536808024847

**Published:** 2008-08-13

**Authors:** Lihua Mao, Yan Chen

**Affiliations:** a School of City Development, University of Jinan, Jinan 250002, People’s Republic of China; b Shandong Blood Center, Jinan 250014, People’s Republic of China

## Abstract

The title compound, C_7_H_10_N_2_O, crystallizes with two independent mol­ecules in the asymmetric unit. The bond lengths and angles in the mol­ecules are within normal ranges. The crystal structure is stabilized by inter­molecular N—H⋯N hydrogen bonds, linking the two independent mol­ecules into hydrogen-bonded dimers.

## Related literature

For related literatures, see: Cai *et al.* (2006 ▶); Yale (1976 ▶). For bond-length data, see: Allen *et al.* (1987 ▶).
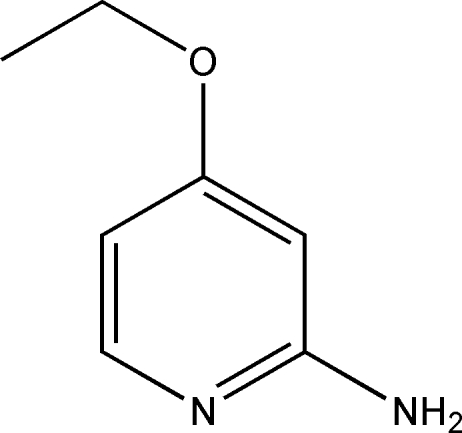


## Experimental

### 

#### Crystal data

C_7_H_10_N_2_O
*M* *_r_* = 138.17
Triclinic, 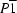

*a* = 9.167 (2) Å
*b* = 9.470 (2) Å
*c* = 9.541 (3) Å
α = 87.716 (3)°
β = 87.714 (4)°
γ = 64.189 (3)°
*V* = 744.8 (3) Å^3^
*Z* = 4
Mo *K*α radiation
μ = 0.09 mm^−1^
*T* = 298 (2) K
0.60 × 0.38 × 0.31 mm

#### Data collection

Bruker SMART CCD area-detector diffractometer
Absorption correction: multi-scan (*SADABS*; Sheldrick, 2004 ▶) *T*_min_ = 0.901, *T*_max_ = 0.974
3749 measured reflections
2582 independent reflections
2110 reflections with *I* > 2σ(*I*)
*R*_int_ = 0.018

#### Refinement

*R*[*F* ^2^ > 2σ(*F* ^2^)] = 0.043
*wR*(*F* ^2^) = 0.123
*S* = 1.02
2582 reflections
182 parameters
H-atom parameters constrained
Δρ_max_ = 0.18 e Å^−3^
Δρ_min_ = −0.16 e Å^−3^

### 

Data collection: *SMART* (Bruker, 2001 ▶); cell refinement: *SAINT* (Bruker, 2001 ▶); data reduction: *SAINT*; program(s) used to solve structure: *SHELXTL* (Sheldrick, 2008 ▶); program(s) used to refine structure: *SHELXTL*; molecular graphics: *SHELXTL*; software used to prepare material for publication: *SHELXTL* and local programs.

## Supplementary Material

Crystal structure: contains datablocks I, global. DOI: 10.1107/S1600536808024847/bx2167sup1.cif

Structure factors: contains datablocks I. DOI: 10.1107/S1600536808024847/bx2167Isup2.hkl

Additional supplementary materials:  crystallographic information; 3D view; checkCIF report

## Figures and Tables

**Table 1:** Hydrogen-bond geometry (Å, °)

*D*—H⋯*A*	*D*—H	H⋯*A*	*D*⋯*A*	*D*—H⋯*A*
N2—H2*B*⋯N1^i^	0.86	2.19	3.029 (2)	164
N4—H4*B*⋯N3^ii^	0.86	2.16	3.013 (2)	173

Symmetry codes: (i) 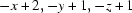; (ii) 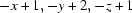.

**Fig. 1.**
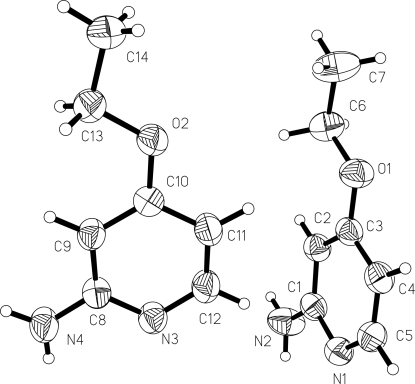
View of the title compound (I), with displacement ellipsoids drawn at the 40% probability level.

**Fig. 2.**
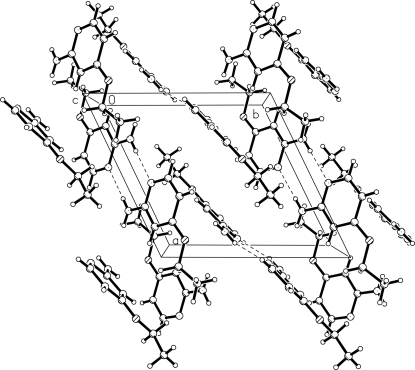
Packing diagram of structure of (I), view along the c axis. Hydrogen bonds are shown as dashed lines.

## supplementary crystallographic information

### Comment

2-Amino-4-ethoxypyridine is a useful intermediate for the synthesis of various heterocyclic compounds (Cai *et al.*, 2006; Yale, 1976). In this paper, we report the crystal structure of the title compound (I). The title compound crystallizes with two independent molecules in the asymmetric unit. All bond lengths are normal (Allen *et al.*, 1987). Intermolecular N—H···N hydrogen bonds link the two independent molecules into hydrogen-bonded dimers. The crystal packing is further stabilized by van der Waals forces.

### Experimental

2-amino-4-chloropyridine (12.9 g, 0.1 mol) and sodium ethoxide (12.8 g, 0.2 mol) were reacted in 100 ml ethanol in a stainless steel bomb at 150°C for 3 h. The desired compound was obtained as a slightly yellow solid in 50% yield (1.9 g). Crystals suitable for X-ray diffraction analysis were obtained by slow evaporation of a solution in a hexane/dichloromethane mixture (1:4 *v*/*v*) at room temperature over a period of one week.

### Refinement

H atoms bonded to N atoms were located in a difference map and refined with distance restraints of N—H = 0.86Å, and with *U*_iso_(H) = 1.2*U*_eq_(N). Other H atoms were positioned geometrically and refined using a riding model, with C—H = 0.93–0.97 Å and with *U*_iso_(H) = 1.2 (1.5 for methyl groups) times *U*_eq_(C).

### Crystal data

**Table:**

C_7_H_10_N_2_O	*Z* = 4
*M**_r_* = 138.17	*F*_000_ = 296
Triclinic, *P*1	*D*_x_ = 1.232 Mg m^−^^3^
Hall symbol: -P 1	Mo *K*α radiation λ = 0.71073 Å
*a* = 9.167 (2) Å	Cell parameters from 1699 reflections
*b* = 9.470 (2) Å	θ = 2.8–23.1º
*c* = 9.541 (3) Å	µ = 0.09 mm^−^^1^
α = 87.716 (3)º	*T* = 298 (2) K
β = 87.714 (4)º	Block, yellow
γ = 64.189 (3)º	0.60 × 0.38 × 0.31 mm
*V* = 744.8 (3) Å^3^	

### Data collection

**Table:**

Bruker SMART CCD area-detector diffractometer	2582 independent reflections
Radiation source: fine-focus sealed tube	2110 reflections with *I* > 2σ(*I*)
Monochromator: graphite	*R*_int_ = 0.018
*T* = 298(2) K	θ_max_ = 25.0º
φ and ω scans	θ_min_ = 2.1º
Absorption correction: multi-scan(SADABS; Sheldrick, 2004)	*h* = −10→10
*T*_min_ = 0.901, *T*_max_ = 0.974	*k* = −10→11
3749 measured reflections	*l* = −11→7

### Refinement

**Table:**

Refinement on *F*^2^	Hydrogen site location: inferred from neighbouring sites
Least-squares matrix: full	H-atom parameters constrained
*R*[*F*^2^ > 2σ(*F*^2^)] = 0.043	*w* = 1/[σ^2^(*F*_o_^2^) + (0.0632*P*)^2^ + 0.1056*P*] where *P* = (*F*_o_^2^ + 2*F*_c_^2^)/3
*wR*(*F*^2^) = 0.123	(Δ/σ)_max_ < 0.001
*S* = 1.02	Δρ_max_ = 0.18 e Å^−^^3^
2582 reflections	Δρ_min_ = −0.16 e Å^−^^3^
182 parameters	Extinction correction: SHELXTL (Sheldrick, 2008), Fc^*^=kFc[1+0.001xFc^2^λ^3^/sin(2θ)]^-1/4^
Primary atom site location: structure-invariant direct methods	Extinction coefficient: 0.040 (5)
Secondary atom site location: difference Fourier map	

### Special details

**Table:**

Geometry. All e.s.d.'s (except the e.s.d. in the dihedral angle between two l.s. planes) are estimated using the full covariance matrix. The cell e.s.d.'s are taken into account individually in the estimation of e.s.d.'s in distances, angles and torsion angles; correlations between e.s.d.'s in cell parameters are only used when they are defined by crystal symmetry. An approximate (isotropic) treatment of cell e.s.d.'s is used for estimating e.s.d.'s involving l.s. planes.
Refinement. Refinement of *F*^2^ against ALL reflections. The weighted *R*-factor *wR* and goodness of fit *S* are based on *F*^2^, conventional *R*-factors *R* are based on *F*, with *F* set to zero for negative *F*^2^. The threshold expression of *F*^2^ > σ(*F*^2^) is used only for calculating *R*-factors(gt) *etc*. and is not relevant to the choice of reflections for refinement. *R*-factors based on *F*^2^ are statistically about twice as large as those based on *F*, and *R*- factors based on ALL data will be even larger.

### Fractional atomic coordinates and isotropic or equivalent isotropic displacement parameters (Å^2^)

**Table:**

	*x*	*y*	*z*	*U*_iso_*/*U*_eq_	
O1	0.59961 (14)	0.31435 (14)	0.17306 (12)	0.0647 (3)	
O2	0.09387 (12)	0.83919 (12)	0.15720 (11)	0.0568 (3)	
N1	0.93140 (16)	0.44443 (15)	0.33834 (14)	0.0559 (4)	
N2	0.8694 (2)	0.3953 (2)	0.56360 (15)	0.0763 (5)	
H2B	0.9418	0.4221	0.5910	0.092*	
H2C	0.8150	0.3666	0.6242	0.092*	
N3	0.44815 (15)	0.88727 (15)	0.37838 (14)	0.0557 (4)	
N4	0.26244 (17)	1.10399 (17)	0.49027 (15)	0.0703 (5)	
H4B	0.3410	1.1061	0.5350	0.084*	
H4C	0.1649	1.1736	0.5056	0.084*	
C1	0.84095 (18)	0.39828 (17)	0.42438 (16)	0.0516 (4)	
C2	0.72722 (18)	0.35088 (17)	0.37698 (16)	0.0521 (4)	
H2A	0.6669	0.3189	0.4401	0.063*	
C3	0.70648 (18)	0.35259 (17)	0.23495 (16)	0.0508 (4)	
C4	0.8027 (2)	0.39719 (19)	0.14405 (17)	0.0576 (4)	
H4A	0.7938	0.3970	0.0473	0.069*	
C5	0.9098 (2)	0.44086 (19)	0.20049 (17)	0.0586 (4)	
H5A	0.9731	0.4707	0.1389	0.070*	
C6	0.4922 (2)	0.2715 (2)	0.25818 (19)	0.0682 (5)	
H6A	0.4340	0.3519	0.3260	0.082*	
H6B	0.5528	0.1732	0.3085	0.082*	
C7	0.3764 (3)	0.2550 (3)	0.1622 (2)	0.0951 (7)	
H7A	0.3023	0.2263	0.2159	0.143*	
H7B	0.4354	0.1752	0.0957	0.143*	
H7C	0.3171	0.3530	0.1132	0.143*	
C8	0.29236 (18)	0.99108 (18)	0.39569 (15)	0.0506 (4)	
C9	0.16603 (18)	0.98351 (18)	0.32351 (16)	0.0511 (4)	
H9A	0.0594	1.0573	0.3380	0.061*	
C10	0.20318 (18)	0.86432 (17)	0.23054 (15)	0.0480 (4)	
C11	0.36506 (19)	0.75665 (18)	0.21075 (17)	0.0568 (4)	
H11A	0.3941	0.6753	0.1483	0.068*	
C12	0.47882 (19)	0.77452 (19)	0.28565 (18)	0.0596 (4)	
H12A	0.5864	0.7029	0.2714	0.072*	
C13	−0.07465 (19)	0.9457 (2)	0.17306 (18)	0.0605 (4)	
H13A	−0.0939	1.0486	0.1348	0.073*	
H13B	−0.1069	0.9557	0.2716	0.073*	
C14	−0.1699 (2)	0.8813 (2)	0.0962 (2)	0.0806 (6)	
H14A	−0.2832	0.9508	0.1049	0.121*	
H14B	−0.1507	0.7799	0.1352	0.121*	
H14C	−0.1371	0.8718	−0.0012	0.121*	

### Atomic displacement parameters (Å^2^)

**Table:**

	*U*^11^	*U*^22^	*U*^33^	*U*^12^	*U*^13^	*U*^23^
O1	0.0665 (7)	0.0772 (8)	0.0598 (7)	−0.0395 (7)	−0.0091 (6)	−0.0017 (6)
O2	0.0522 (6)	0.0562 (7)	0.0601 (7)	−0.0206 (5)	−0.0068 (5)	−0.0106 (5)
N1	0.0577 (8)	0.0564 (8)	0.0557 (8)	−0.0264 (7)	−0.0021 (6)	−0.0038 (6)
N2	0.1046 (12)	0.0994 (12)	0.0514 (8)	−0.0687 (11)	−0.0128 (8)	0.0064 (8)
N3	0.0489 (7)	0.0531 (8)	0.0580 (8)	−0.0148 (6)	−0.0073 (6)	−0.0054 (6)
N4	0.0526 (8)	0.0734 (10)	0.0741 (10)	−0.0143 (7)	−0.0102 (7)	−0.0284 (8)
C1	0.0562 (9)	0.0425 (8)	0.0532 (9)	−0.0186 (7)	−0.0054 (7)	−0.0003 (6)
C2	0.0557 (9)	0.0466 (9)	0.0535 (9)	−0.0219 (7)	−0.0009 (7)	0.0002 (7)
C3	0.0501 (8)	0.0418 (8)	0.0572 (9)	−0.0164 (7)	−0.0049 (7)	−0.0040 (7)
C4	0.0648 (10)	0.0591 (10)	0.0486 (9)	−0.0263 (8)	−0.0019 (7)	−0.0045 (7)
C5	0.0624 (10)	0.0625 (10)	0.0538 (9)	−0.0303 (8)	0.0052 (7)	−0.0040 (7)
C6	0.0628 (10)	0.0736 (12)	0.0730 (11)	−0.0342 (9)	−0.0093 (9)	0.0062 (9)
C7	0.0799 (14)	0.1224 (19)	0.1023 (16)	−0.0620 (14)	−0.0346 (12)	0.0319 (14)
C8	0.0507 (9)	0.0497 (9)	0.0464 (8)	−0.0167 (7)	−0.0048 (6)	−0.0018 (7)
C9	0.0450 (8)	0.0487 (9)	0.0523 (8)	−0.0130 (7)	−0.0030 (6)	−0.0040 (7)
C10	0.0515 (8)	0.0476 (8)	0.0451 (8)	−0.0216 (7)	−0.0043 (6)	0.0023 (6)
C11	0.0557 (9)	0.0493 (9)	0.0603 (9)	−0.0172 (8)	0.0001 (7)	−0.0118 (7)
C12	0.0481 (9)	0.0513 (9)	0.0693 (10)	−0.0116 (7)	−0.0018 (8)	−0.0083 (8)
C13	0.0528 (9)	0.0603 (10)	0.0650 (10)	−0.0207 (8)	−0.0045 (8)	−0.0074 (8)
C14	0.0627 (11)	0.0813 (13)	0.1024 (15)	−0.0335 (10)	−0.0108 (10)	−0.0174 (11)

### Geometric parameters (Å, °)

**Table:**

O1—C3	1.3460 (19)	C5—H5A	0.9300
O1—C6	1.433 (2)	C6—C7	1.490 (3)
O2—C10	1.3518 (18)	C6—H6A	0.9700
O2—C13	1.4364 (19)	C6—H6B	0.9700
N1—C1	1.336 (2)	C7—H7A	0.9600
N1—C5	1.342 (2)	C7—H7B	0.9600
N2—C1	1.361 (2)	C7—H7C	0.9600
N2—H2B	0.8600	C8—C9	1.400 (2)
N2—H2C	0.8600	C9—C10	1.378 (2)
N3—C12	1.340 (2)	C9—H9A	0.9300
N3—C8	1.3434 (19)	C10—C11	1.397 (2)
N4—C8	1.3545 (19)	C11—C12	1.360 (2)
N4—H4B	0.8600	C11—H11A	0.9300
N4—H4C	0.8600	C12—H12A	0.9300
C1—C2	1.398 (2)	C13—C14	1.491 (2)
C2—C3	1.374 (2)	C13—H13A	0.9700
C2—H2A	0.9300	C13—H13B	0.9700
C3—C4	1.394 (2)	C14—H14A	0.9600
C4—C5	1.355 (2)	C14—H14B	0.9600
C4—H4A	0.9300	C14—H14C	0.9600
			
C3—O1—C6	119.52 (13)	C6—C7—H7B	109.5
C10—O2—C13	118.61 (11)	H7A—C7—H7B	109.5
C1—N1—C5	116.21 (13)	C6—C7—H7C	109.5
C1—N2—H2B	120.0	H7A—C7—H7C	109.5
C1—N2—H2C	120.0	H7B—C7—H7C	109.5
H2B—N2—H2C	120.0	N3—C8—N4	116.04 (14)
C12—N3—C8	116.47 (13)	N3—C8—C9	122.94 (14)
C8—N4—H4B	120.0	N4—C8—C9	121.01 (14)
C8—N4—H4C	120.0	C10—C9—C8	118.52 (14)
H4B—N4—H4C	120.0	C10—C9—H9A	120.7
N1—C1—N2	115.68 (14)	C8—C9—H9A	120.7
N1—C1—C2	123.24 (14)	O2—C10—C9	125.11 (14)
N2—C1—C2	121.06 (15)	O2—C10—C11	115.89 (13)
C3—C2—C1	118.51 (14)	C9—C10—C11	118.98 (14)
C3—C2—H2A	120.7	C12—C11—C10	117.94 (14)
C1—C2—H2A	120.7	C12—C11—H11A	121.0
O1—C3—C2	125.72 (14)	C10—C11—H11A	121.0
O1—C3—C4	115.54 (14)	N3—C12—C11	125.14 (15)
C2—C3—C4	118.74 (14)	N3—C12—H12A	117.4
C5—C4—C3	118.19 (15)	C11—C12—H12A	117.4
C5—C4—H4A	120.9	O2—C13—C14	107.85 (14)
C3—C4—H4A	120.9	O2—C13—H13A	110.1
N1—C5—C4	125.08 (15)	C14—C13—H13A	110.1
N1—C5—H5A	117.5	O2—C13—H13B	110.1
C4—C5—H5A	117.5	C14—C13—H13B	110.1
O1—C6—C7	107.17 (15)	H13A—C13—H13B	108.4
O1—C6—H6A	110.3	C13—C14—H14A	109.5
C7—C6—H6A	110.3	C13—C14—H14B	109.5
O1—C6—H6B	110.3	H14A—C14—H14B	109.5
C7—C6—H6B	110.3	C13—C14—H14C	109.5
H6A—C6—H6B	108.5	H14A—C14—H14C	109.5
C6—C7—H7A	109.5	H14B—C14—H14C	109.5
			
C5—N1—C1—N2	177.01 (15)	C12—N3—C8—N4	179.60 (15)
C5—N1—C1—C2	−1.1 (2)	C12—N3—C8—C9	0.8 (2)
N1—C1—C2—C3	−0.4 (2)	N3—C8—C9—C10	0.0 (2)
N2—C1—C2—C3	−178.37 (15)	N4—C8—C9—C10	−178.74 (15)
C6—O1—C3—C2	1.9 (2)	C13—O2—C10—C9	1.6 (2)
C6—O1—C3—C4	−178.15 (14)	C13—O2—C10—C11	179.93 (13)
C1—C2—C3—O1	−178.26 (14)	C8—C9—C10—O2	177.69 (14)
C1—C2—C3—C4	1.8 (2)	C8—C9—C10—C11	−0.6 (2)
O1—C3—C4—C5	178.35 (14)	O2—C10—C11—C12	−178.04 (14)
C2—C3—C4—C5	−1.7 (2)	C9—C10—C11—C12	0.4 (2)
C1—N1—C5—C4	1.2 (2)	C8—N3—C12—C11	−1.1 (3)
C3—C4—C5—N1	0.2 (3)	C10—C11—C12—N3	0.5 (3)
C3—O1—C6—C7	173.22 (16)	C10—O2—C13—C14	−173.07 (15)

### Hydrogen-bond geometry (Å, °)

**Table:**

*D*—H···*A*	*D*—H	H···*A*	*D*···*A*	*D*—H···*A*
N2—H2B···N1^i^	0.86	2.19	3.029 (2)	164
N4—H4B···N3^ii^	0.86	2.16	3.013 (2)	173

Symmetry codes: (i) −*x*+2, −*y*+1, −*z*+1; (ii) −*x*+1, −*y*+2, −*z*+1.
